# Residual Feed Intake and Rumen Metabolism in Growing Pelibuey Sheep

**DOI:** 10.3390/ani12050572

**Published:** 2022-02-24

**Authors:** Carlos Arce-Recinos, Nadia Florencia Ojeda-Robertos, Ricardo Alfonso Garcia-Herrera, Jesús Alberto Ramos-Juarez, Ángel Trinidad Piñeiro-Vázquez, Jorge Rodolfo Canul-Solís, Luis Enrique Castillo-Sanchez, Fernando Casanova-Lugo, Einar Vargas-Bello-Pérez, Alfonso Juventino Chay-Canul

**Affiliations:** 1 División Académica de Ciencias Agropecuarias, Universidad Juárez Autónoma de Tabasco, Carretera Villahermosa-Teapa, Km 25, R/A, La Huasteca 2ª Sección, Villahermosa 86280, Tabasco, Mexico; carlos.arce@colpos.mx (C.A.-R.); nadia.ojeda@ujat.mx (N.F.O.-R.); ricardo.garcia@ujat.mx (R.A.G.-H.); 2 Colegio de Postgraduados, Campus Tabasco, Periférico Carlos A. Molina, Km 3.5, Carretera Cárdenas-Huimanguillo, H. Cárdenas 86500, Tabasco, Mexico; ramosj@colpos.mx; 3 Tecnológico Nacional de México, Instituto Tecnológico de Conkal, Avenida Tecnológico s/n, Conkal 97345, Yucatán, Mexico; angel.pineiro@itconkal.edu.mx; 4 Tecnológico Nacional de México, Instituto Tecnológico de Tizimín, Tizimín 97702, Yucatán, Mexico; jorge.canul@ittizimin.edu.mx (J.R.C.-S.); luis.castillo@ittizimin.edu.mx (L.E.C.-S.); 5 Tecnológico Nacional de Mexico, Instituto Tecnológico de la Zona Maya, Othón P. Blanco 77965, Quintana Roo, Mexico; fernando.cl@zonamaya.tecnm.mx; 6 Department of Veterinary and Animal Sciences, Faculty of Health and Medical Sciences, University of Copenhagen, Grønnegårdsvej 3, DK-1870 Frederiksberg C, Denmark

**Keywords:** residual feed intake, volatile fatty acids, methane, Pelibuey, rumen fermentation

## Abstract

**Simple Summary:**

This study determined residual feed intake (RFI), volatile fatty acid (VFA) production and enteric methane (CH_4_) from growing Pelibuey sheep. In this case, 12 non-castrated Pelibuey were classified as low, medium, and high RFI. Efficient lambs (low-RFI) had lower intakes of dry matter, organic matter, crude protein and neutral detergent fiber. Those lambs produced less CH_4_. Feed intake of low RFI lambs was approximately 16% lower (*p* < 0.05) while growth rate was not significantly different. Their average energy loss, expressed as CH_4_ production per kilogram of metabolic weight, was 17% lower (*p* < 0.05).

**Abstract:**

This study was carried out to evaluate the residual feed intake (RFI), volatile fatty acid (VFA) production and enteric methane (CH_4_) from growing Pelibuey sheep. In this case, 12 non-castrated Pelibuey with an initial average live weight (LW) of 21.17 ± 3.87 kg and an age of 3 months, were housed in individual pens and fed a basal diet with 16% of crude protein and 11 MJ ME for 45 days. Dry matter intake (DMI) was measured and the daily weight gain (DWG) was calculated using a linear regression between the LW and experimental period. Mean metabolic live weight (LW^0.75^) was calculated. RFI was determined by linear regression with DWG and LW^0.75^ as independent variables. Lambs were classified as low, medium, and high RFI. Feed efficiency was determined as DWG/DMI. For determining rumen pH, ammonia nitrogen concentration NH_3_-N)_,_ and VFA, ruminal fluid was obtained using an esophageal probe on day 40. Feed intake of low RFI lambs was approximately 16% lower (*p* < 0.05) while growth rate was not significantly different. Their average energy loss, expressed as CH_4_ production per kilogram of metabolic weight, was 17% lower (*p* < 0.05).

## 1. Introduction

Livestock production contributes significantly to greenhouse gas emissions, with an estimated 8.1 gigatons of CO_2_-eq emitted in 2010, with methane (CH_4_) accounting for 50% of emissions from livestock [1]. In addition to being a polluting gas with a warming potential 25 times higher than CO_2_, it has been determined that the production of enteric CH_4_ represents a loss of 2–12% of the gross energy ingested [2]. This is due to the low quality of pastures with contents of crude protein (CP) < 75 and acid detergent fiber (ADF) > 70, which reduces forage digestibility and increases the amount of energy lost [3], thereby reducing the metabolizable energy destined for productive behavior [4]. For this reason, strategies are currently being sought to increase the efficiency in the use of available resources in the tropics and improve the sustainability of production systems.

In recent years, various tools have been sought to help explain, predict and select animals with greater efficiency in the use of feed and energy consumed. The evaluation of residual feed intake (RFI) is a tool that determines animals’ feed efficiency [5], and therefore, RFI is currently used as a quantitative parameter for genetic improvement in cattle [6], since it helps to reduce production costs. Residual feed intake is the difference between feed intake and expected feed intake for a given weight and productive level [7]. Recent studies in hair sheep indicated that efficient lambs (low RFI) eat less feed and have growth rates similar compared to inefficient animals [8,9,10].

In addition, the RFI contributes to reducing the negative effects of livestock production systems on the environmental impact, since it has been documented that those animals with lower RFI emit less methane per unit of dry matter intake (DMI) [11,12,13] and they improve the efficiency in the use of energy and nitrogen [14,15] in addition, animals with lower RFI require less maintenance energy.

This favors the mitigation of CO_2_, N_2_O, CH_4_ emissions and reduces the environmental impact of livestock production. The selection of lambs with better efficiency after weaning will have the benefit that these animals will be more efficient and will produce less methane in the later stages of growth [13]. In addition, sheep with lower RFI will reduce the use of imported grains at high prices that compete with food security and increase production costs and reduce the profitability of production systems in the tropical region.

In Mexico, RFI is being evaluated in livestock production systems; therefore, generating information on enteric CH_4_ emissions from efficient animals is of vital importance. However, the studies that relate the RFI with the molar proportion of volatile fatty acids (VFA) to estimate the production of CH_4_ in growing Pelibuey sheep are scarce. In general, it is unknown in the tropical region what the methane emission factor is per unit of dry matter intake (Ym) or per kg of live weight increased in hair sheep. Therefore, the objective of this study was to evaluate the RFI and the proportion of volatile fatty acids (VFA) to estimate CH_4_ production in growing Pelibuey sheep under humid tropical conditions.

## 2. Materials and Methods

### 2.1. Experimental Site

The animals included in the present study were managed in compliance with the regulations for the use and care of animals intended for research and were approved by the Ethics Committee on Animal Use and Animal Care of the Universidad Juarez Autonoma de Tabasco (SUBINVTAB.CP.-105/18). The study was carried out at the facilities of the Centro de Integración Ovina del Sureste (CIOS), located in Villahermosa, Tabasco, Mexico (17°50′ N, 93°23′ W). The region’s climate is warm with rains all year round (Af) and the average annual temperature is 27.8 °C [16].

### 2.2. Animals and Experimental Design

In this case, 12 male Pelibuey sheep were used with an initial mean live weight (LW) of 21.17 ± 3.87 kg (mean ± SD) with an age of 3 months. Animals were housed in individual pens (2 × 2 m) provided with feeders and drinkers. Before starting the experimental phase, the animals were dewormed with Moxidectin (Pfizer, São Paulo, Brazil) at a rate of 0.2 mg/kg LW and ADE vitamins (1 mL per 10 kg of LW) were applied intramuscularly. The experiment lasted 60 days, with a 15-day diet and 45-day for data collection. The latter was divided into three periods of 15 days.

Lambs were fed ad libitum daily at 08:00 and 15:00 with a total mixed ration. The amount of feed offered per day was adjusted to guarantee at least 15% rejection. The diet was formulated to meet the nutritional requirements of growing lambs with 20 kg and daily weight gains of 250 g (Table 1). Table 2 shows the chemical composition of the diet [17].

### 2.3. Productive Traits

The amount of feed offered and rejected was recorded daily to calculate dry matter intake (DMI), organic matter intake (OMI), crude protein intake (CPI), and neutral detergent fiber intake (NDFI). Lambs were weighed every 15 days, before offering a morning meal. The daily weight gain (DWG) was estimated as the linear regression slope between the LW and the experimental period. Feed conversion (FC) was calculated as the relationship between DMI and DWG. Residual feed intake (RFI) was estimated using regression between DMI, metabolic live weight (LW^0.75^), and DWG according to Koch et al. [18]. The resulting equation was used to calculate the estimated DMI (DMIe).

DMIe = −0.4806 (±0.207) + 0.0002 (±0.0.001) × DWG + 0.1313 (±0.019) × LW^0.75^ (r^2^ = 0.91)

Subsequently, by the difference between the observed DMI and the DMIe, the residuals were obtained for each lamb. The mean and standard deviation (SD) of the residuals were calculated, and the lambs were classified as efficient or low-RFI (<0.5 SD below the mean), medium-RFI (±0.5 SD of the mean), and high-RFI (>0.5 SD above the mean).

### 2.4. Determination of pH, Ammonia Nitrogen Concentration (NH_3_-N), Volatile Fatty Acid Production, and Methane Emissions

On day 40 of the test, before offering the morning food, samples of ruminal fluid were taken by intragastric tube [19] to determine pH, concentrations of NH_3_-N, and volatile fatty acids (VFA). Immediately, the ruminal fluid samples were filtered with double gauges to measure pH with a portable potentiometer (HANNA^®^ Instruments, Woonsocket, RI, USA).

Ammonia nitrogen concentration was determined by the Phenol-Hypochlorite method described by Taylor [20]. For VFA analysis, 4 mL of ruminal fluid were taken and conserved in 1 mL of a deproteinizing solution composed of metaphosphoric acid and 3-methyl valeric acid. The VFA concentrations were determined with the gas chromatography technique (Hewlett-Packard, 5890 series III, Mundelein, IL, USA) according to that described by Gonzáles et al. [21]. The chromatograph was equipped with a flame ionization detector (FID) and a 30 m × 0.53 mm HP-FFAP column. The injector and detector temperature was 200 °C. Methane production in the rumen was estimated using volatile fatty acid molar ratios as suggested by Moss et al. [22].

### 2.5. Chemical Analysis

The samples of feed offered and refused were analyzed for contents of DM, ash, crude protein, and ether extract. DM was determined after drying at 105 °C, and ash after combustion at 550 °C. Crude fat was extracted for 6 h with petroleum ether, whereas the Kjeldahl method was used to determine nitrogen (N) [23]. CP was calculated as N × 6.25. The contents of a neutral detergent (NDF) and acid detergent fiber (ADF) were determined according to the methods of Van Soest et al. [24]. ADF and lignin were determined using fiber bags and using an ANKOM 220 Fibre Analyzer (ANKOM Technology Corporation, Macedon, NY, USA).

### 2.6. Statistical Analysis

Data on nutrient intake, productive performance, rumen fermentation parameters, and estimation of methane production were compared by efficiency groups (low-RFI, medium-RFI, and high-RFI) using a completely randomized design, and each animal was considered an experimental unit. For the statistical analysis, the PROC GLM procedure of SAS (v9.3, SAS Inst. Inc., Cary, NC, USA) was used, using the statistical model described below:

$$Y_{i}=µ+\beta_{i}+\varepsilon$$

in which *Yi* is the response variable of the *i*-th animal, µ is the population mean, *β_i_* is the effect of the RFI or RIG class (low, medium, high) of the *i*-th animal and, ε is the residual error. The least-squares means were calculated and compared using the Tukey test, and they were considered statistically significant when *p* < 0.05.

## 3. Results and Discussion

Lambs had an average DMI of 1.15 ± 0.069 kg/d and a DWG of 216 ± 8.677 g/d. Low-RFI lambs consumed 84 g/d less feed than expected (*p* < 0.001), while high-RFI lambs consumed 77 g/d more than expected (Table 3). No differences were observed in DMI, however, a numerical difference of 150 g/d was observed between efficient and inefficient lambs. Lambs with low-RFI had a lower (*p* ≤ 0.05) DMI, OMI, CPI, and NDFI when it was expressed in g/kg of LW^0.75^, and had a lower percentage of DMI in relation to LW^0.75^, compared to lambs with high-RFI (Table 3). These results agree with that reported by Arce-Recinos et al. [10] who observed in Pelibuey lambs with low-RFI lower DMI (34.1 vs. 40.2 g), OMI (31.7 vs. 37.4 g), CPI (5.4 vs. 6.2 g), NDFI (13.2 vs. 15.9 g) and a lower percentage of DMI (8.3 vs. 9.6%) than lambs with high-RFI when feed intake was expressed in relation to LW^0.75^. On the other hand, the difference of 150 g/d between efficient and inefficient lambs is in accordance with that reported in hair sheep, which ranged from 160 to 190 g/d [8,9,10].

The LW^0.75^, initial LW, final LW, and DWG did not differ between lambs grouped by RFI (*p* > 0.05, Table 3). This is explained because RFI is independent of productive level and body size [18]. Although efficient lambs had a lower DMI compared to inefficient lambs, they showed a similar growth rate, which is explained by the fact that individuals with low-RFI have a lower metabolizable energy requirement for maintenance [25]. Or they have a different ruminal microbial composition, which means that animals with lower RFI have better fermentation and digestibility of the diet, so they use the nitrogen and energy consumed more efficiently. No differences were observed in the FC between the efficiency groups because no differences were found in the DMI and the DWG, similar results were reported by Arce-Recinos et al. [10] in Pelibuey sheep. On the other hand, this result differs from that reported by Lima et al. [8] and Rocha et al. [26], who observed a better FC in the crossbred lambs 1/2 Dorper × 1/2 Santa Inês and 3/4 Texel × 1/4 Pantaneira with low-RFI (4.43 vs. 5.15 kg, 4.18 vs. 5.00 kg), respectively.

The concentration of VFA, pH, N-NH_3,_ and production of CH_4_ are presented in Table 3. The molar proportions of acetate, propionate, butyrate, iso-valerate, iso-butyrate, valerate, and the Acetate: Propionate ratio were similar (*p* > 0.05) among lambs grouped by RFI (Table 4). The pH and N-NH_3_ concentrations did not differ (*p* > 0.05) between the efficiency groups (Table 4). These results agree with that reported by Arce-Recinos et al. [10], who did not observe differences in the fermentation parameters between efficient and inefficient Pelibuey lambs. This is attributed to the fact that the type of feed consumed was the same in both groups, which does not generate a change in fermentation and the molar proportion of volatile fatty acids between animals with high and low RFI.

Except for CH_4_ production expressed in L LW^0.75^/d, methane production did not differ among lambs classified by RFI (Table 4). Lambs with low-RFI had a lower (*p* < 0.05) production of CH_4_ when standardized in L/LW^0.75^/d compared to lambs with high-RFI, observing a difference of 0.24 L/LW^0.75^/d. The results of this study agree with that reported by Muro-Reyes et al. [27], who reported that the methane estimated by equations was lower in low RFI Rambouillet sheep breed (0.021 vs. 0.027 kg/d, and 0.025 vs. 0.032 kg/d) than in inefficient sheep. Likewise, it agrees with the results documented in cattle [11,12], where animals with low-RFI produce less methane (1.28 vs 1.71 L/kg of LW^0.75^, 142.3 vs. 190.2 g/d, respectively).

RFI has been used as a selection criterion in beef cattle breeding programs. The estimated heritability of this trait is moderate (0.27–0.58) [18,28,29] and independent of growth, it does not negatively affect other economically important traits such as the quality of meat produced [30]. Likewise, RFI reduces the environmental impact of livestock, since animals with low RFI tend to produce less CH_4_ per unit of DM [11,12], due to lower consumption of DM and better efficiency in the use of energy [15]. That is why the RFI represents one of the mitigation strategies for CO_2_ and CH_4_ emissions).

CH_4_ is considered a by-product of carbohydrate fermentation in the rumen and is considered a loss of ingested gross energy and in ruminants, it represents between 2–12% [2]. It has been documented that CH_4_ production is related to feed efficiency and feed quality, therefore inefficient individuals with higher dry matter intake produce more CH_4_ [11,15] having greater energy loss and causing lower profitability of the production system. In this sense, rumen microorganisms play a very important role in fermentation, methane production, and feed efficiency. It has been reported that the methanogenic communities in high-RFI animals are more diverse, presenting a high prevalence of *Methanosphaera stadtmaniae* and *Methanobrevibacter* sp. [31]. Kittelman et al. [32] showed that there are differences in the ruminal bacterial community that is linked to high or low CH_4_ emission in sheep. In sheep with low methane emission, the ruminotypes were characterized by species such as *Fibrobacter* spp., *Kandleria vitulina, Olsenella* spp., *Prevotella bryantii*, and *Sharpea azabuensis*, while in queen sheep with high methane emission, a high abundance of species such as *Ruminococcus*, *Ruminococcaceae*, *Lachnospiraceae*, *Catabacteriaceae*, *Coprococcus*, *Clostridiales*, *Prevotella*, *Bacteroidales*, *Alphaproteobacteria.*

On the other hand, it has been reported that the rumen of efficient lamb’s harbours more abundant and diverse microbial communities, with a higher Firmicutes: Bacteroidetes ratio that is related to energy metabolism [33], and a greater presence of specialized microorganisms in propionate production [14], which favors a better performance in meat production [15]. Furthermore, a recent study has reported that the diversity and relative abundance indices of microbial taxa are heritable (h^2^ ≥ 0.15) and are associated with the characteristic of feeding efficiency in the host [34]. Therefore, it is important to characterize the ruminal microbiome (protozoa and bacteria) in low and high RFI animals. In addition, recent studies indicate that in animals with a low RFI (efficient animals) there are types of ruminal bacteria that are associated with this characteristic [31,35,36]. In steers with low RFI, differences were detected in the genes of *Succinivibrio* sp., while in animals with high RFI, genes of *Robinsoniella* sp. [35]. In lambs, *Ruminococcus flavefaciens* and *Ruminococcus albus* were present in greater abundance (*p* < 0.001) in lambs with high RFI (3.2 times higher for *R. flavefaciens*; 1.5 times higher for *R. albus*) compared to animals with low RFI [36]. The knowledge of the interrelationships between the ruminal bacterial communities and the host animal could be a tool that would allow selecting animals with a natural (innate) capacity to emit less ruminal methane.

It is important to highlight that the small sample size was one limitation of the present study. However, this study could open the possibility to continue elucidating the relationship of RFI between performance, carcass, and meat quality in hair sheep breeds and their crosses in tropical regions. Therefore, future studies may be considered the minimal number of observations and include studying different breeds, body weights, and different physiological or growth stages.

## 4. Conclusions

Feed intake of low RFI lambs was approximately 16% lower (*p* < 0.05) while the growth rate was not significantly different. Their average energy loss expressed as CH_4_ production per kilogram of metabolic weight, was 17% lower (*p* < 0.05). This indicates that animals with lower RFI may improve the profitability of the system.

## Figures and Tables

**Table 1:** Ingredients of the diet used for feeding lambs.

Ingredients	% Dry Matter
Ground sorghum grain	29.3
Star grass hay	27.0
Soybean meal	14.5
Wheat bran	11.0
Coconut meal	8.9
Molasses	6.5
Vitamin and mineral premix *	1.2
Calcium carbonate	1.0
Sodium bicarbonate	0.6
Total	100

* Vitamin and mineral premix (in 1 kg): 40 g P, 60 g Ca, 20 g Mg, 0.0003 mg Se, 0.0005 mg Co, 0.1 mg Mn; 0.003 mg I, 0.1 mg Zn, 0.0002 mg Cu, 33.6 mg vitamin A, 0.55 mg vitamin D and 557.1 mg vitamin E.

**Table 2:** Chemical composition of the diet used for feeding lambs (dry matter basis).

Dry matter, %	89.2
Crude protein, %	16.0
Neutral detergent fiber, %	40.0
Acid detergent fiber, %	18.5
Lignin, %	3.9
Ash, %	8.3
Ether extract, %	2.5
Metabolizable energy (MJ/kg DM)	11.0

**Table 3:** Nutrient intake and productive performance of Pelibuey lambs classified as low, medium, and high residual feed intake.

Variable	Residual Feed Intake			*p*-Value
	Low (*n* = 3)	Medium (*n* = 6)	High (*n* = 3)	
Residual feed intake (g/d)	−83.9 ± 25.27 ^c^	4.4 ± 10.99 ^b^	76.6 ± 20.97 ^a^	0.001
Dry matter intake (kg/d)	0.99 ± 0.12	1.26 ± 0.12	1.14 ± 0.08	0.313
Dry matter intake (% LW^0.75^)	8.6 ± 0.38 ^b^	9.7 ± 0.27 ^a,b^	9.9 ± 0.26 ^a^	0.036
Dry matter intake (g/kg/d LW^.75^)	85.5 ± 3.79 ^b^	97.0 ± 2.68 ^a,b^	99.4 ± 2.58 ^a^	0.032
Organic matter intake (g/kg/d LW^0.75^)	77.8 ± 3.60	88.0 ± 2.56	89.9 ± 2.38	0.057
Crude protein intake (g/kg/d LW^0.75^)	16.3 ± 0.60 ^b^	18.8 ± 0.54 ^a^	19.5 ± 0.49 ^a^	0.005
Neutral detergent fiber intake (g/kg/d LW^0.75^)	30.5 ± 1.17 ^b^	35.0 ± 1.00 ^a^	36.2 ± 0.92 ^a^	0.010
Daily weight gain (g/d)	205 ± 22.9	229 ± 11.9	208 ± 12.4	0.481
Feed conversion (kg DMI/kg of DWG)	4.96 ± 0.71	5.46 ± 0.32	5.53 ± 0.46	0.696
Metabolic weight (LW^0.75^)	11.55 ± 0.85	12.84 ± 0.88	11.45 ± 0.55	0.435
Initial live weight	19.79 ± 1.65	23.11 ± 2.18	18.62 ± 0.89	0.279
Final live weight	30.60 ± 2.64	34.93 ± 2.74	30.07 ± 1.52	0.371

^a,b,c^ Means in the same row with different superscripts are different (*p* < 0.05).

**Table 4:** Rumen fermentation parameters and estimation of methane production in Pelibuey lambs classified as low, medium, and high residual feed intake.

Parameters	Residual Feed Intake			*p*-Value
	Low (*n* = 3)	Medium (*n* = 6)	High (*n* = 3)	
pH	6.75 ± 0.05	6.83 ± 0.07	6.94 ± 0.12	0.369
N-NH_3_ (mg/dL)	23.9 ± 1.98	23.3 ± 5.19	38.3 ± 6.11	0.149
Volatile fatty acids (mol/100 mol)				
Acetate	59.7 ± 0.12	58.4 ± 3.05	59.3 ± 1.67	0.951
Propionate	21.0 ± 3.99	21.0 ± 3.17	18.8 ± 1.52	0.874
Butyrate	14.4 ± 0.87	15.6 ± 1.36	15.8 ± 0.97	0.731
Iso-valerate	2.12 ± 0.26	2.06 ± 0.17	3.12 ± 0.14	0.055
Iso-butyrate	1.34 ± 0.09	1.52 ± 0.25	1.67 ± 0.15	0.634
Valerate	1.42 ± 0.27	1.30 ± 0.30	1.16 ± 0.31	0.866
Acetate: Propionate	3.09 ± 0.66	3.16 ± 0.66	3.20 ± 0.33	0.994
CH_4_ (mM/L)	26.8 ± 2.97	26.7 ± 2.38	25.8 ± 2.79	0.966
CH_4_ (L/d)	37.3 ± 4.35	47.2 ± 4.44	42.9 ± 2.95	0.316
CH_4_ (L/kg DMI)	37.6 ± 0.01	37.6 ± 0.06	37.6 ± 0.03	0.547
CH_4_ (L/LW^0.75^/d)	3.21 ± 0.15 ^b^	3.65 ± 0.11 ^a,b^	3.75 ± 0.09 ^a^	0.037

^a,b^ Means in the same row with different superscripts are different (*p* < 0.05).

## Data Availability

The data presented in this study are available on request from the corresponding author.
